# H3F3A-G34R mutant high grade neuroepithelial neoplasms with glial and dysplastic ganglion cell components

**DOI:** 10.1186/s40478-019-0731-5

**Published:** 2019-05-20

**Authors:** Felipe Andreiuolo, Tomo Lisner, Jozef Zlocha, Christof Kramm, Arend Koch, Brigitte Bison, Albane Gareton, Marc Zanello, Andreas Waha, Pascale Varlet, Torsten Pietsch

**Affiliations:** 1 Department of Neuropathology, DGNN Brain Tumor Reference Center, University of Bonn Medical Center, Bonn, Germany; 2 Department of Neuropathology, Sainte-Anne Hospital, Paris, France; 3 Department of Pediatrics, Clinic Chemnitz, Chemnitz, Germany; 4 Division of Pediatric Hematology and Oncology, University Medical Center Göttingen, Göttingen, Germany; 5 Department of Neuropathology, Berlin Institute of Health, Charité - Universitätsmedizin Berlin, Freie Universität Berlin, Berlin, Germany; 6 Institute for Diagnostic and Interventional Neuroradiology, University Hospital Würzburg, Würzburg, Germany; 7 Department of Neurosurgery, Sainte-Anne Hospital, Paris, France

**Keywords:** Anaplastic ganglioglioma, Histone H3, Mutation, G34R, Neuroepithelial tumor, Glioblastoma

## Abstract

The recently described malignant neuro-epithelial tumors with histone *H3F3A* point mutations at G34 (NET-H3-G34) occur most often in cerebral hemispheres of teenagers and young adults, and have a generally adverse prognosis. These tumors have been histologically classified as glioblastoma or primitive neuroectodermal tumor (PNET) in the past, and have not been defined as a separate entity in the revised WHO classification of tumors of the CNS 2016. Here, we report two cases of NET-H3-G34 with glial and dysplastic ganglion cell components affecting teenagers. Patients were treated with surgery and radiochemotherapy with temozolomide. One patient underwent partial resection and deceased 21 months after diagnosis, while the other patient is alive without evidence of disease 15 months after total resection. So far, a dysplastic ganglion cell component has not been described in NET-H-G34, and its presence raises a possible relation to (anaplastic) gangliogliomas. Genome-wide copy number analysis did not provide unequivocal evidence that these tumors represent anaplastic variants of gangliogliomas, as opposed to NET-H3-G34. Our observations expand the morphologic spectrum of NET-H3-G34. Further cases of NET-H3-G34 with dysplastic ganglion cells should be clinically followed to find differences or similarities in their biological behavior, as compared to NET-H3-G34 and anaplastic gangliogliomas.

## Introduction

The recently described malignant neuro-epithelial tumors with histone *H3F3A* point mutations at G34 (NET-H3-G34) occur most often in cerebral hemispheres of teenagers and young adults, and have a generally adverse prognosis, with reported median PFS and OS of 8–9 months and 12–22 months [3, 8]. They typically show an undifferentiated phenotype with a small blue-cell component, a glioblastoma-like astrocytic component or a mixture of the two, and therefore have been histologically classified as glioblastoma or primitive neuroepithelial tumor in the past [2, 8]. These tumors have not been defined as a separate entity in the recently revised WHO classification. We report two cases of H3F3A-G34R mutant, high grade neuroepithelial neoplasms with glial and dysplastic ganglion cell components. To our knowledge, the presence of dysplastic ganglion cells in tumors carrying *H3F3A* G34 mutations has not yet been reported.

## Case presentation

**Fig. 1:**
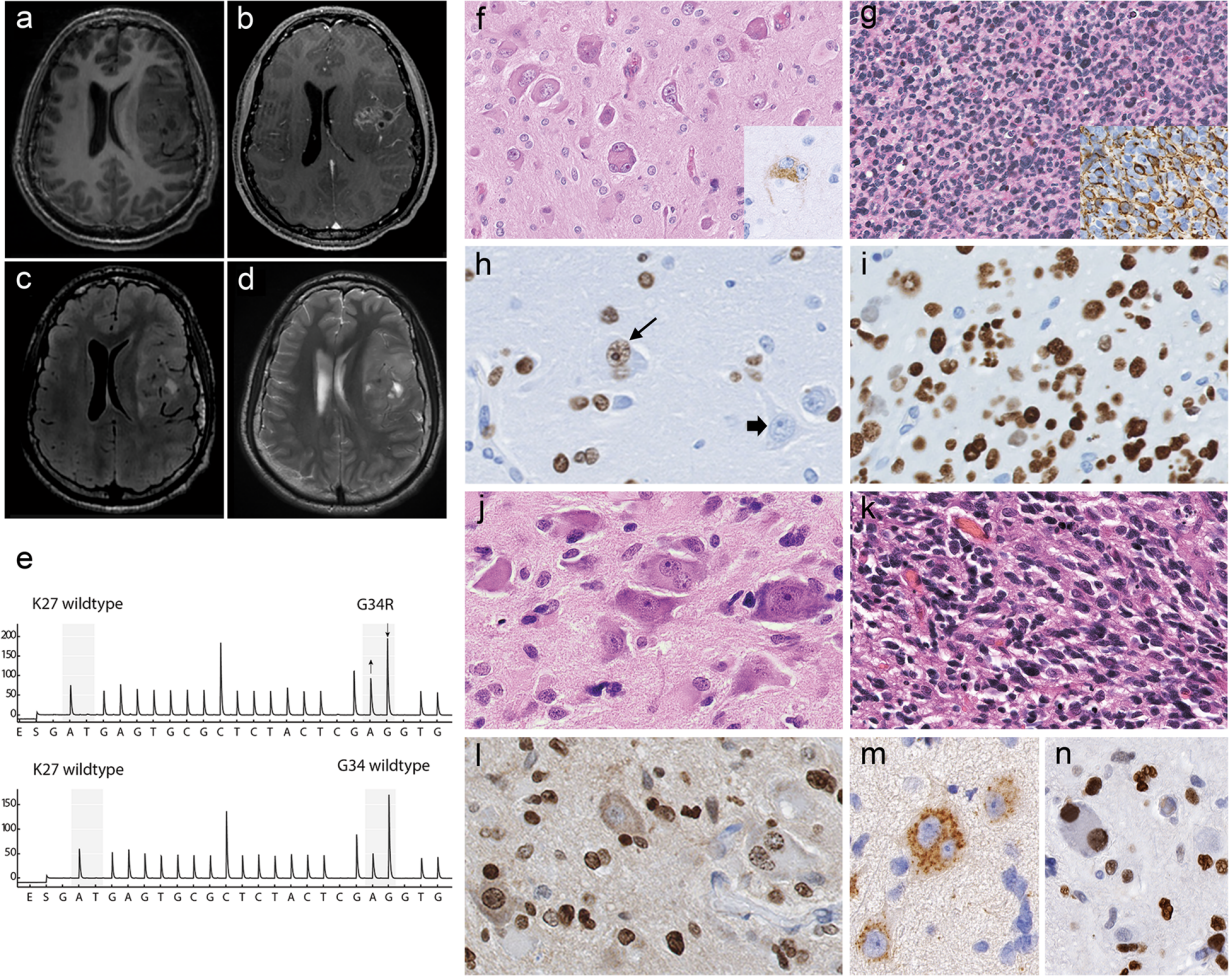
**a**-**c**, pre-operative MR of case 1 T1-weighted pre- (**a**) and post- (**b**) gadolinium images showed a fronto-insular mass with hypointensity and heterogeneous enhancement. On FLAIR- (**c**) and T2-weighted images (**d**), the tumor displayed a solid component with slight hyperintensity and signs of surrounding edema. The mutation was confirmed by pyrosequencing (**e**). The upper part shows the mutation in tumor tissue as compared to the control sample, shown below. **f**-**i**, histopathology of case 1. Hematoxylin phloxin safranine stain revealed a tumor with both neuronal and glial components. Large multinucleated neurons (**f**), positive for chromogranin A (insert), and a major glial diffuse component (**g**), positive for GFAP (insert) were found. H3-G34R was positive in neoplastic neuronal cells (h, thin arrow) and in neoplastic glial cells (**i**), but negative in non-neoplastic neurons (h, thick arrow). **j**-**n**, histopathology of case 2 The tumor displayed abundant binucleated ganglionic cells (**j**) as well as glial tumor cells (**k**), both positive for H3-G34R (**l**). The dysplastic ganglion cells strongly expressed chromogranin (**m**) and showed nuclear accumulation of p53 (**n**)

Case 1 affected a 16-year-old male patient with significant weight loss in 1 year, headaches and visual impairment developing over 2 months. Magnetic resonance imaging (MRI) demonstrated a left-sided fronto-temporo-insular mass, hypointense on T1 (Fig. 1a) with inhomogeneous contrast enhancement (Fig. 1b) and significant mass effect. The tumor showed a solid component with a slightly hyperintense signal and signs of a small surrounding edema on FLAIR (Fig. 1c) and T2-weighted images (Fig. 1d). After partial resection the tumor progressed rapidly, despite radiochemotherapy with temozolomide. The patient deceased 21 months after surgery.

Case 2 involved a 14-year-old male patient presenting with headaches for 8 weeks. Computed tomography scans showed a partially hyperdense tumor with calcifications and slight contrast enhancement. MRI revealed a right occipital cortical/−subcortical tumor with a cystic component, measuring 5.3 × 4.6 × 6.8 cm^3^, extending to the falx, hyperintense on FLAIR-weighted images, slightly hypointense in T1 with small hyperintense spots, compatible with calcifications and blood. The tumor was sharply demarcated from the surrounding brain parenchyma, which presented no significant signs of edema. The patient underwent total resection and radiochemotherapy with temozolomide, and 15 months after surgery was asymptomatic, without radiological evidence of residual or progressive disease.

**Fig. 2:**
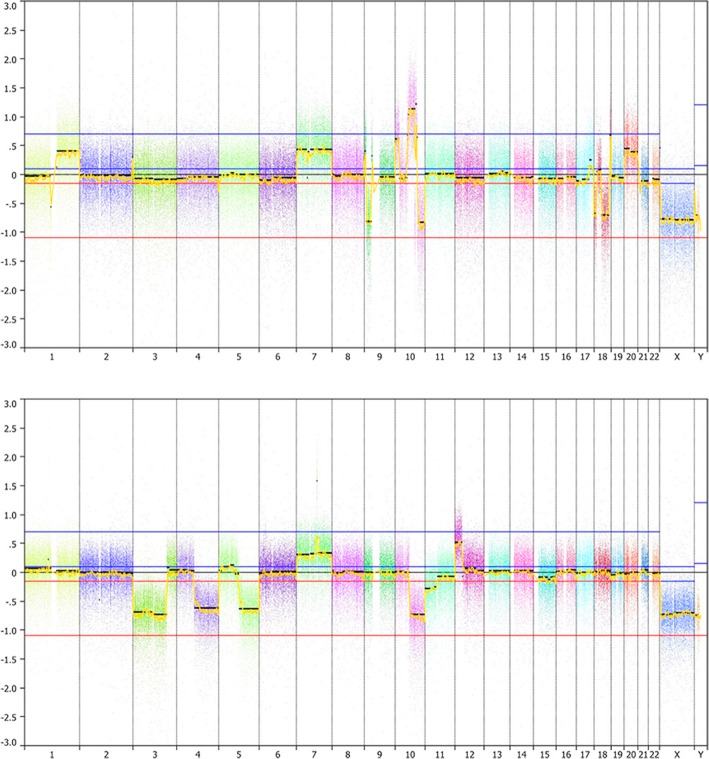
Molecular inversion probe assay plots from cases 1(top) and 2 (bottom) are shown. Chromosomes are illustrated by different colours

Histologically, both tumors showed mixed neuronal and glial components (Fig. 1f, g, j, k), with similar immunophenotypes. The neuronal component consisted of large bi- or multi-nucleated neurons, (Fig. 1f, j) positive for chromogranin A (Fig. 1f insert; j, m), also displaying cytoplasmic expression of synaptophysin. The predominant glial component was composed of diffusely infiltrating small cells (Fig. 1g, k) expressing glial fibrillary acidic protein (Fig. 1 g insert), but not Olig2. Perineuronal satellitosis, perivascular clustering and subpial infiltration were present in case 1 only. Eosinophilic granular bodies were absent. Mitotic activity was high. Vascular proliferation was only present in case 2; palisading necrosis was observed in both cases. The proliferation activity (Ki-67 staining) was high. IDH1-R132H, BRAF-V600E and H3-K27 M proteins were not detectable. ATRX was lost in both neuronal and glial tumor cells. Both cases displayed CD34-positive satellite cells. H3-G34R immunostaining [4] was positive in neoplastic neuronal cells (Fig. 1h, l) and neoplastic glial cells (Fig. 1i, l), but negative in entrapped neurons. p53 was strongly accumulated in the nuclei of both glial and neuronal tumor cells in case 2 (Fig. 1n), but was negative in case 1. Pyrosequencing confirmed the presence of a *H3F3A* G34R mutation in both cases (Fig. 1e). Classification by DNA methylation profiling was in agreement with H3G34 tumors (not shown). Molecular Inversion Probe analysis revealed among other alterations gains of chromosome 7 and 10q losses in both tumors (Fig. 2). Case 1 showed gain of chromosome 1q and loss of 9p including the *CDKN2A* locus, as well as indication of chromothripsis of chromosome 10. Case 2 displayed amplification of *CDK6* and loss of chromosome 3q and 4q (Fig. 2).

## Discussion and conclusions

A ganglion cell differentiation in NET-H3-G34 is not surprising, as the specific pattern of H3K36me3 binding in these tumors upregulates genes particularly involved in neuronal morphogenesis and differentiation, such as *MYCN* and *DLX6*, among others [1]. However, dysplastic ganglion cells are a hallmark of (anaplastic) gangliogliomas. The question remains, whether the tumors described here represent a variant of “NET-H3-G34” or a biologically distinct variant of anaplastic ganglioglioma (AGG). AGGs (WHO grade III) are rare glioneuronal tumors with a generally unfavorable prognosis. Among adults, a median PFS of 8 months and median OS of 24.7 months were reported [12]. Children with AGG seem to have a better prognosis, with 63% 5-year-PFS and 88% 5-year-OS in a series of 8 cases [6].

The radiological findings described for gangliogliomas and “NET-H3-G34” do not enable a clear distinction between them [11, 13]. Cytogenetically, a distinction is also difficult, as very few cases of AGG have been described in the literature. Case 1 displayed chromosome 9p losses, including *CDNK2A*, which have been described in a case of AGG [5], and are frequently seen in glioblastomas [9], as well as in 14% of NET-H3-G34 [8]. Case 2 showed loss of chromosome 3q and 4q, reported in 67–70% of NET-H3-G34, and also a *CDK6* amplification seen in 10% of NET-H3-G34 [8]. These alterations have not been described in AGGs so far. The presence of chromosome 7 gain seen in the two cases is a typical feature of glioblastomas [9], and also frequently observed in NET-H3-G34 [8]. However, this finding does not completely rule out an AGG, as chromosome 7 gains seem to be a frequent cytogenetic alteration observed in 21% of gangliogliomas [5].

Histone *H3F3A*-K27 M mutations have been detected in AGG [7, 13], but so far *H3F3A*-G34 mutations have not been reported in AGG. One should keep in mind that the histological spectrum of mutations associated with tumor entities is often wider than initially described – a good example is the growing list of tumors with *BRAF*-V600E mutations or *histone H3*-K27 mutations. Occurrence of a certain mutation, therefore, may not per se define a tumoral entity. On the other hand, ganglion cell differentiation is not sufficient for the diagnosis of a ganglioglioma, as this feature has been documented in different glial tumors, including oligodendroglioma IDH-mutant and 1p/19q codeleted [10]. However, in the cases described here the ganglionic cell component is clearly dysplastic. The term “neuroepithelial tumors with histone H3-G34 mutations” proposed by Korshunov et al. [8] seems adequate, as the morphological spectrum of these tumors is wide. Therefore, our observations further expand the morphologic spectrum of NET-H3-G34 beyond glioblastomas with or without PNET-like components. More patients with such rare NET-H3-G34 with dysplastic ganglion cells should be clinically followed to find differences or similarities in their biological behavior, as compared to other NET-H3-G34 and AGG.

## Abbreviations

AGG: Anaplastic ganglioglioma
FLAIR: Fluid-attenuated inversion recovery
MRI: Magnetic resonance imaging
NET-H3-G34: Neuro-epithelial tumors with histone *H3F3A* point mutations at G34
PNET: Primitive neuroectodermal tumor
WHO: World Health Organization
